# Recurrence of Gestational Diabetes Mellitus: To Assess Glucose Metabolism and Clinical Risk Factors at the Beginning of a Subsequent Pregnancy

**DOI:** 10.3390/jcm10204794

**Published:** 2021-10-19

**Authors:** Grammata Kotzaeridi, Julia Blätter, Daniel Eppel, Ingo Rosicky, Veronica Falcone, Gabriela Adamczyk, Tina Linder, Gülen Yerlikaya-Schatten, Karen Weisshaupt, Wolfgang Henrich, Andrea Tura, Christian S. Göbl

**Affiliations:** 1 Department of Obstetrics and Gynecology, Medical University of Vienna, 1090 Vienna, Austria; grammata.kotzaeridi@meduniwien.ac.at (G.K.); julia.blaetter@gmx.de (J.B.); daniel.eppel@meduniwien.ac.at (D.E.); ingo.rosicky@meduniwien.ac.at (I.R.); veronica.falcone@meduniwien.ac.at (V.F.); gabi-alexa@live.de (G.A.); tina.linder@meduniwien.ac.at (T.L.); guelen.yerlikaya-schatten@meduniwien.ac.at (G.Y.-S.); 2 Clinic of Obstetrics, Charité-Universitätsmedizin Berlin, Corporate Member of Freie Universität Berlin, Humboldt-Universität zu Berlin, and Berlin Institute of Health, 13353 Berlin, Germany; karen.weisshaupt@charite.de (K.W.); wolfgang.henrich@charite.de (W.H.); 3 Metabolic Unit, CNR Institute of Neuroscience, 35127 Padova, Italy; andrea.tura@cnr.it

**Keywords:** gestational diabetes mellitus, GDM recurrence, hyperglycemia, pregnancy, epidemiology

## Abstract

Women with a history of gestational diabetes mellitus (GDM) are at high risk of developing hyperglycemia in a subsequent pregnancy. This study aimed to assess parameters of glucose metabolism at the beginning of a subsequent pregnancy in women with a history of GDM. This prospective cohort study included 706 women who had at least one previous pregnancy (120 with prior GDM and 586 without GDM history). All study participants received a broad risk evaluation and laboratory testing at the beginning of a subsequent pregnancy and were followed up until delivery to assess GDM status, risk factors for GDM recurrence, and pregnancy outcomes. Women with a history of GDM exhibited lower insulin sensitivity and subtle impairments in β-cell function associated with subclinical hyperglycemia already at the beginning of a subsequent pregnancy compared to women without GDM history. This was associated with a markedly increased risk for the later development of GDM (OR: 6.59, 95% CI 4.34 to 10.09, *p* < 0.001). Early gestational fasting glucose and HbA1c were identified as the most important predictors. Mothers with a history of GDM showed marked alterations in glucose metabolism at the beginning of a subsequent pregnancy, which explains the high prevalence of GDM recurrence in these women.

## 1. Introduction

Women with a history of gestational diabetes mellitus (GDM) are predisposed to develop hyperglycemia in a subsequent pregnancy [1]. Previous studies identified a recurrence rate of approximately 50%, which is associated with several risk factors such as ethnic origin, maternal body mass index (BMI) as well as treatment with insulin and delivery of an offspring with macrosomia in the previous pregnancy [2,3]. This high prevalence of GDM recurrence can be explained by pathophysiological mechanisms: the development of GDM is closely related to subclinical impairments of insulin action and β-cell function, which eventually emerge as manifest hyperglycemia when insulin sensitivity physiologically decreases in the second and third trimester of pregnancy [1,4]. Although the majority of patients return to normal glucose tolerance when insulin sensitivity improves after delivery, these subtle defects in glucose metabolism persist and can be unmasked in a subsequent pregnancy (leading to a recurrence of GDM) or by unhealthy lifestyle, aging, and obesity (leading to type 2 diabetes) in the patient’s later life [1,5]. Moreover, assessment of postpartum glucose tolerance via OGTT is often neglected due to inadequate counseling and poor patient compliance, leading to a failure in identifying persistent impairments of glucose metabolism and act upon it [6,7].

The characterization of glucose metabolism at the beginning of a subsequent pregnancy is therefore of clinical importance in order to identify subgroups at high risk of developing GDM or obstetric complications as well as to provide timely interventions. However, there is only limited information available on this topic. Therefore, this study aimed to assess metabolic characteristics including laboratory parameters and indices of glucose metabolism at the beginning of a subsequent pregnancy of women with a history of GDM. Moreover, the specific characteristics of subgroups, where glycemic status between the two pregnancies differed, were assessed and risk factors for the development of GDM in a subsequent pregnancy were evaluated.

## 2. Materials and Methods

### 2.1. Study Design and Participants

In this prospective cohort study, we included a total of 1132 pregnant women without pre-existing diabetes, who attended our pregnancy outpatient clinic (Department of Obstetrics and Gynecology, Medical University of Vienna) between January 2016 and July 2019. Nulliparous women (*n* = 426) were excluded from this report, leading to an effective sample size of 706 patients. The study participants received a broad risk evaluation at 13 weeks of gestation (IQR: 12 to 14), including the assessment of pregestational and current body mass index (BMI), maternal age, parity, obstetric history (including GDM in previous pregnancies), family history of diabetes and ethnicity. In addition, we performed routine laboratory testing at the baseline visit to assess fasting plasma glucose, insulin and C-peptide, lipids and glycated hemoglobin A1c (HbA1c) concentrations. The study participants underwent universal GDM screening by use of a 75 g 2 h oral glucose tolerance test (OGTT) in the late second or early third trimester, which was implemented in Austrian maternity care in 2011. Thereby, GDM was diagnosed according to the International Diabetes in Pregnancy Study Groups (IADPSG) recommendations (i.e., fasting glucose ≥92 mg/dL; 1 h post-load glucose ≥180 mg/dL; 2 h post-load glucose ≥153 mg/dL) [8], which were adopted by the WHO in 2013 [9]. In patients with fasting glucose ≥92 mg/dL before 24 weeks of gestation, the presence of GDM was verified by either early OGTT or self-monitored blood glucose in accordance with our national guidelines [10]. All laboratory parameters were measured according to the standard laboratory methods at our certified Department of Medical and Chemical Laboratory Diagnostics (http://www.kimcl.at, accessed on 11 February 2021): Plasma glucose was measured by the hexokinase method (coefficient of variation, CV: 1.3%). The levels of insulin (CV: 4% to 7%) and C-peptide (CV: 3% to 4%) were measured by chemiluminescence immunoassays. HbA1c was assessed by high-performance liquid chromatography (IFCC standardized and DCCT aligned with CV: 1.8%). Offspring data was assessed after delivery. Thereby, calculations of age and sex-adjusted percentiles of the Austrian population were based on an analysis of the local growth standard curves. Large for gestational age (LGA) was defined as birth weight above the 90th percentile, whereas macrosomia was defined as birth weight greater than 4000 g. The study was approved by the Ethics Committee of the Medical University of Vienna and performed in accordance with the Declaration of Helsinki. All participants gave written informed consent.

### 2.2. Calculations

The degree of insulin sensitivity was assessed by the quantitative insulin sensitivity check index calculated from insulin (QUICKIi) and C-peptide (QUICKIc) [11]. Moreover, we used a modified insulinogenic index (IGI = FCP (ng/mL)/FPG (mg/dL)), whereby FCP is fasting C-peptide and FPG is fasting glucose, to estimate insulin secretion [12]. The product of IGI × FI^−1^ (whereby FI is fasting insulin) was used as the modified oral disposition index (DI) to estimate β-cell function (i.e., the capability of the pancreatic β-cells to adapt for the amount of insulin resistance) from fasting parameters [13].

### 2.3. Statistical Analysis

Continuous variables were summarized by the mean ± standard deviation or as median and interquartile ranges (IQR) in case of skewed distribution and compared by Welch’s *t*-test, analysis of variance or rank-based inference, as appropriate. Categorical variables were summarized by counts and percentages and compared by use of a binomial logistic model. When more than two groups were compared, we used Tukey’s HSD to achieve a 95% coverage probability. Odds ratios and 95% Confidence Intervals (95% CI) were additionally calculated for binary outcomes. Discrimination (i.e., the ability of a risk factor to separate pregnant women with the disease from those without the disease) was assessed by C-statistics, resulting in values between 50 and 100, whereby a value of 100 means perfect discrimination. Random decision forests were created by the conditional inference framework (cforest) to derive measures of variable importance, which is calculated as the average difference in predictive accuracy before and after random permutation of the values of a predictor variable overall (i.e., 1 × 10^5^) trees [14,15]. Statistical analysis was performed with R (version 4.0.2, R Foundation for Statistical Computing, Vienna, Austria) and contributing packages [16]. A two-sided *p*-value of ≤0.05 was considered statistically significant.

### 2.4. Results

**Figure 1:**
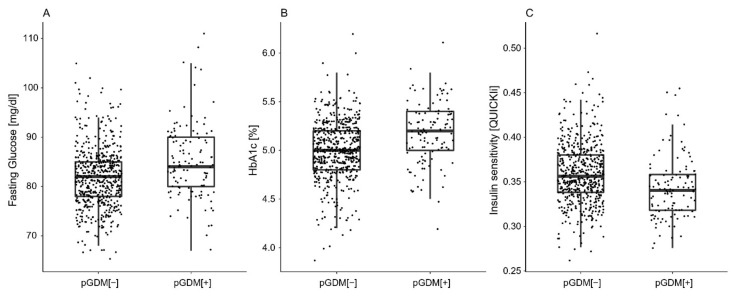
Glucometabolic parameters in early pregnancy for women without history of a pregnancy with GDM (pGDM [−]) vs. patients with history of GDM in previous pregnancy (pGDM [+]). Fasting glucose (**A**), HbA1c (**B**), and insulin sensitivity (**C**).

A total of 120 women with a history of GDM in at least one of their previous pregnancies were identified, whereby 23 (19.2%) had more than one pregnancy with GDM and 56 (46.7%) received insulin treatment in a previous pregnancy. Descriptive characteristics of patients with a history of GDM (pGDM [+]) compared to women who remained normal glucose tolerant in previous pregnancies (pGDM [−]) are provided in Table 1. In general, pGDM [+] mothers showed to have higher BMI and more often had a family history of diabetes. Moreover, they tended to have increased triglyceride concentrations and an unfavorable glucometabolic profile at the beginning of a subsequent pregnancy (Figure 1). This included elevated fasting glucose and HbA1c levels, as well as impairments in insulin sensitivity and β-cell function, which were associated with higher glucose levels during the OGTT and an increased risk for recurrence of GDM in the current pregnancy (OR: 6.59, 95% CI 4.34 to 10.09, *p* < 0.001). The increased risk for GDM remained significant after adjustment for pregestational BMI and maternal age (OR: 5.91, 95% CI 3.85 to 9.12, *p* < 0.001) as well as in a sensitivity analysis including women with one previous pregnancy only (OR: 6.89, 95% CI 3.62 to 13.3, *p* < 0.001). The results remained unchanged after excluding patients who had their last pregnancy before the implementation of universal OGTT testing in Austria (Supplementary Materials Table S1).

As a further analysis, we classified patients according to their GDM status in the previous and current pregnancy. Thereby, 489 (69.3%) women showed normal glucose tolerance in all pregnancies (NGT-pGDM [−]), 52 (7.4%) women showed normal glucose tolerance in the current pregnancy but had GDM at least in one previous pregnancy (NGT-GDM [+]), 97 (13.7%) women developed GDM in the current pregnancy but remained normal glucose tolerant in previous pregnancies (GDM-pGDM [−]), and 68 (9.6%) women had GDM in prior and current gestations (GDM-pGDM [+]). The characteristics of the subgroups are provided in Table 2. The GDM-pGDM [+] subgroup showed higher HbA1c levels compared to all other groups. Of note, both subgroups with GDM in the current pregnancy (GDM-pGDM [−] and GDM-pGDM [+]), but also the NGT-pGDM [+] group exhibited a higher degree of insulin resistance in early pregnancy, which was further associated with hyperinsulinemia. In addition, an impaired β-cell function was observed in GDM-pGDM [−] and GDM-pGDM [+] women compared to normal glucose tolerant women without a history of GDM.

**Figure 2:**
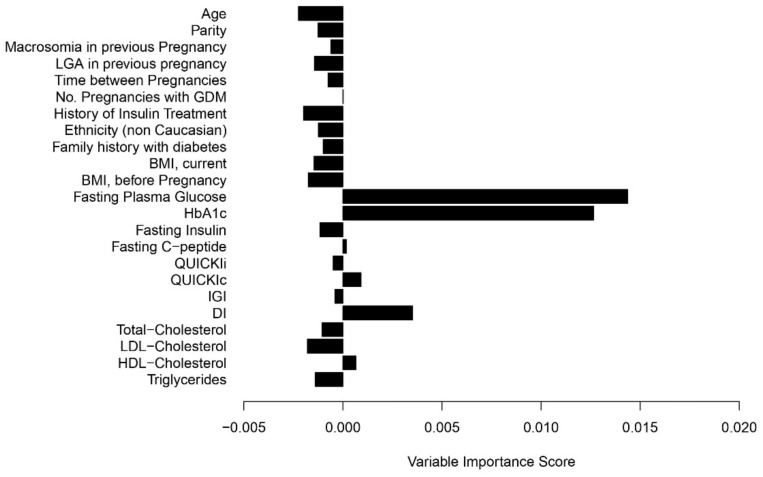
Variable importance scores for the prediction of GDM in a subsequent pregnancy for women with history of GDM.

Analyses of early gestational risk factors for the development of GDM in the current pregnancy are provided in Table 3. For this purpose, we limited the data to the 120 pGDM [+] women (68 patients who developed GDM and 52 who remained NGT) and found that fasting glucose, HbA1c as well as fasting insulin and DI in early gestation reached significance in univariable analyses. In addition, random forest analysis was performed to quantify variable importance. As shown in Figure 2, fasting plasma glucose, HbA1c and DI achieved the highest variable importance scores. Their discrimination in terms of C-statistics was moderate to fair (FPG: 66.8, 95% CI: 57.1 to 76.6; HbA1c: 66.6, 95% CI: 57.0 to 76.5; DI: 63.0, 95% CI: 52.4 to 73.7).

Details of neonatal biometry and pregnancy outcomes are provided in the Supplementary Materials (Table S2). Of note, infants of mothers with a history of GDM showed significantly increased birth weight percentiles compared to women with normal glucose tolerance in previous gestations (mean difference: 6.0, 95% CI: 0.45 to 11.6). However, this difference lost significance after adjustment for GDM in the current pregnancy (*p* = 0.123). No differences were observed in birth weight percentiles (NGT-pGDM [−]: 45.0 ± 3.0 vs. NGT-pGDM [+]: 52.6 ± 28.0 vs. GDM-pGDM [−]: 49.8 ± 26.4 vs. GDM-pGDM [+]: 51.2 ± 27.1; *p* > 0.25) or in other obstetric complications when patients were grouped according to their GDM status in the previous or current pregnancy.

## 3. Discussion

This study aimed to assess clinical characteristics and parameters of glucose metabolism at the beginning of a subsequent pregnancy in women with a history of GDM. We found that mothers with GDM in a previous pregnancy had an unfavorable glucometabolic risk profile with higher BMI and family history of diabetes compared to women who remained normal glucose tolerant during their previous pregnancies. Most importantly, women with prior GDM showed lower insulin sensitivity and subtle impairments in β-cell function already at an early stage of a subsequent pregnancy, which was associated with elevated fasting glucose and HbA1c levels and a markedly increased risk of GDM recurrence.

While this is, to our knowledge, the first study providing a detailed metabolic characterization at the beginning of a subsequent pregnancy in women with prior GDM, the prevalence of GDM recurrence has been addressed in previous studies and meta-analyses. A systematic review identified a recurrence rate of approximately 48%, which is somehow lower than that observed in our study (56%) [3]. However, the reported prevalence of GDM recurrence varied widely across different studies, ranging from 29% to 80%. Thereby, ethnic origin (39% GDM recurrence rate was reported for non-Hispanic white women vs. 56% for other ethnicities) and parity (40% for primiparous vs. 73% for multiparous women) were identified as potential sources of heterogeneity [3]. In another systematic review, Schwartz et al. concluded that the prevalence of GDM recurrence was related to maternal body composition (BMI and weight gain between pregnancies), as well as delivery of macrosomic offspring and insulin therapy in the previous pregnancy in addition to ethnicity and parity [2]. Conversely, traditional risk factors showed inferior accuracy compared to laboratory parameters in our study. This might be explained by the application of different diagnostic criteria for GDM. While most previous studies used the National Diabetes Data Group (1979) criteria, we used the most recent IADPSG recommendations for GDM classification. However, it was mentioned by some authors that, due to the increased GDM prevalence associated with the IADPSG approach [17,18], the effect size of several risk factors might decrease, which is in line with our observations [2]. As a sensitivity analysis, we limited our analysis to patients who received their first GDM diagnosis after the implementation of universal GDM screening according to IADPSG recommendations in Austria. However, both recurrence rate and clinical risk factors did not change significantly.

While the use of laboratory parameters such as fasting glucose or HbA1c for confirmation or exclusion of GDM before 24 weeks of gestation is controversially discussed, their potential role as early pregnancy risk markers for the later development of hyperglycemia is gaining more and more attention [19,20,21,22,23]. In this context, we found that laboratory parameters such as fasting glucose and HbA1c achieved the highest variable importance scores with regard to predicting the development of hyperglycemia in the specific subgroup of mothers with GDM in the previous pregnancy. Thereby, fasting glucose and HbA1c showed moderate to fair prognostic accuracy. The tendency towards subclinical hyperglycemia in women with a history of GDM was accompanied by decreased insulin sensitivity. Moreover, the modified disposition index was lower compared to women without GDM in a previous pregnancy, indicating a subclinical defect in β-cell function as well. This is consistent with the pathophysiological mechanisms underlying the development of GDM. Of note, the disposition index was predicting the later development of GDM in addition to fasting glucose and HbA1c. We are not aware of other studies addressing laboratory parameters and pathophysiological components of glucose metabolism at the beginning of a subsequent pregnancy in mothers with GDM history. However, a retrospective Korean study found that patients with recurrent GDM had increased fasting glucose concentrations and impaired insulin action (assessed by the Homeostasis Model Assessment of Insulin Resistance, HOMA-IR) two months after index pregnancy, which is in line with our results in early gestation of women with subsequent pregnancy [24]. In this context, a recent meta-analysis indicated that interpregnancy weight loss can reduce the prevalence of GDM recurrence in overweight or obese mothers with a history of GDM [25]. Weight loss in the interconception period may improve insulin sensitivity and support an adequate adaptation to physiological demands in a subsequent pregnancy, resulting in lower glucose levels and lower risk for GDM and associated complications [26]. Unfortunately, there is little information about the role of interconception care in women with a history of GDM as randomized controlled trials are missing [27]. However, based on the available data we hypothesize that specific lifestyle modification after pregnancy with GDM may reduce the high recurrence rate as well as impairments in glucose metabolism, that were observed at the start of a subsequent pregnancy in our study.

Boghossian et al. observed that GDM in a previous pregnancy increases the risk for large for gestational age offspring in a subsequent pregnancy even without GDM recurrence [28]. The authors hypothesized that this might be caused by subclinical impairment of glucose metabolism in those patients, who did not meet the diagnostic criteria for GDM. In line with this consideration, we found that women with a history of GDM but normal glucose tolerance in the current pregnancy had higher BMI and a lower degree of insulin sensitivity compared to women who exhibited normal glucose tolerance in both pregnancies. In addition, we observed higher birth weight percentiles in offspring of mothers with a history of GDM. Of note, the significance was lost when patients were grouped according to their GDM status in the previous or the current pregnancy. Interestingly, it has been reported that for women at high risk for GDM early screening together with prompt treatment can prevent GDM-related acceleration of fetal growth, which is detectable as early as 20 weeks of gestation [29,30,31].

Advantages and limitations of this study need to be addressed: The large sample size and the prospective design is a major advantage of this study, that provides novel information regarding glucose metabolism at the beginning of a subsequent pregnancy in women with a history of GDM. However, due to the large sample size, insulin sensitivity and secretion were evaluated by fasting measurements, which have some limitations compared to dynamic tests [32,33].

We conclude that women with a history of GDM showed alterations in glucose metabolism, including impairments in insulin sensitivity and β-cell function as well as subclinical hyperglycemia already at an early stage of a subsequent pregnancy. This was associated with a high recurrence rate of the disorder. Lastly, there is a need for further research with regard to improving insulin sensitivity in the interconception period and consequently lowering the risk of recurrence of hyperglycemia in high-risk patients with a history of GDM.

## Figures and Tables

**Table 1:** Characteristics of the study sample.

Variable	pGDM [−] (*n* = 586)	pGDM [+] (*n* = 120)	*p*-Value
Age (years)	32.2 ± 5.3	32.9 ± 4.8	0.217
Parity (≥2)	251 (42.8)	73 (60.8)	<0.001
Parity (≥3)	99 (16.9)	29 (24.2)	0.061
Time between pregnancies (years)	3.0 (2.0–6.0)	4.0 (2.0–5.0)	0.643
Ethnicity (non Caucasian)	151 (25.8)	36 (30.0)	0.339
Family history (1st and 2nd degree)	261 (44.5)	71 (59.2)	0.004
BMI, current (kg/m^2^)	25.2 ± 5.5	27.7 ± 5.8	<0.001
BMI, before pregnancy (kg/m^2^)	25.7 ± 5.4	28.3 ± 5.7	<0.001
Multiple pregnancy	50 (8.5)	3 (2.5)	0.032
Triglycerides, early pregnancy (mg/dL)	118 ± 46.2	129 ± 45.9	0.010
Total-cholesterol, early pregnancy (mg/dL)	189 ± 35.5	191 ± 31.4	0.456
LDL-cholesterol, early pregnancy (mg/dL)	95.3 ± 28.3	98.7 ± 26.3	0.216
HDL-cholesterol, early pregnancy (mg/dL)	69.8 ± 15.7	66.8 ± 14.7	0.053
FPG, early pregnancy (mg/dL)	81.8 ± 6.1	85.1 ± 7.6	<0.001
HbA1c, early pregnancy (%)	5.01 ± 0.29	5.17 ± 0.32	<0.001
HbA1c, early pregnancy (mmol/mol)	31.2 ± 3.2	33.0 ± 3.4	<0.001
Fasting insulin, early pregnancy (µU/mL)	7.9 (5.4–11.2)	10.2 (7.0–16.2)	<0.001
Fasting C-Peptide, early pregnancy (ng/mL)	1.60 (1.30–2.00)	2.00 (1.50–2.50)	<0.001
QUICKIi, early pregnancy (dimensionless) × 10^2^	35.9 ± 3.4	34.3 ± 3.5	<0.001
QUICKIc, early pregnancy (dimensionless) × 10^2^	47.6 ± 3.8	45.4 ± 3.8	<0.001
IGI, early pregnancy (ng/mg)	2.1 ± 0.76	2.4 ± 0.90	<0.001
DI, early pregnancy (ng mg^−1^ (µU/mL)^−1^) × 10^2^	24.6 (20.1–30.4)	21.3 (17.7–26.1)	<0.001
GDM, current pregnancy	97 (16.6)	68 (56.7)	<0.001
OGTT Glucose 0 min (mg/dL)	81.3 ± 8.6	86.9 ± 12.7	<0.001
OGTT Glucose 60 min (mg/dL)	131.1 ± 32.9	159.7 ± 39.3	<0.001
OGTT Glucose 120 min (mg/dL)	106.5 ± 24.1	121.9 ± 31.3	<0.001

Data are the mean ± SD or median (IQR) and count (%) for women without history of a pregnancy with GDM (pGDM [−]) vs. patients with history of GDM in previous pregnancy (pGDM [+]). BMI: body mass index; FPG: fasting plasma glucose; HbA1c: glycated hemoglobin A1c; QUICKIi: quantitative insulin sensitivity check index from insulin (QUICKIi) and C-peptide (QUICKIc); IGI: insulinogenic index; DI: disposition index.

**Table 2:** Characteristics of subgroups categorized according to history of GDM in previous pregnancy and current GDM status.

Variable	NGT-pGDM [−]	NGT-pGDM [+]	GDM-pGDM [−]	GDM-pGDM [+]
	(*n* = 489)	(*n* = 52)	(*n* = 97)	(*n* = 68)
Age (years)	32.0 ± 5.3	32.4 ± 4.9	33.3 ± 5.4	33.2 ± 4.8
Parity (≥2)	205 (41.9)	31 (59.6)	46 (47.4)	42 (61.8) *
Parity (≥3)	77 (15.7)	9 (17.3)	22 (22.7)	20 (29.4) *
Time between pregnancies (years)	3.0 (2.0–6.0)	4.0 (2.0–5.0)	4.0 (2.0–8.0) *	3.0 (2.0–5.0)
Ethnicity (non Caucasian)	121 (24.7)	12 (23.1)	30 (30.9)	24 (35.3)
Family history (1st and 2nd degree)	206 (42.1)	31 (59.6)	55 (56.7) *	40 (58.8) *
BMI, current (kg/m^2^)	25.2 ± 5.5	27.4 ± 5.1 *	27.9 ± 4.7 *	29.0 ± 6.0 *
BMI, before pregnancy (kg/m^2^)	24.8 ± 5.5	26.9 ± 5.4 *	27.2 ± 4.8 *	28.2 ± 6.1 *
Multiple pregnancy	38 (7.8)	3 (6.8)	12 (12.4)	0 (0.0)
Triglycerides, early pregnancy (mg/dL)	113 ± 42.6	126 ± 45.4	142 ± 55.8 *	131 ± 46.5 *
Total-cholesterol, early pregnancy (mg/dL)	189 ± 35.6	192 ± 29.7	190 ± 35.4	190 ± 32.8
LDL-cholesterol, early pregnancy (mg/dL)	95.0 ± 28.3	101.1 ± 24.1	96.7 ± 28.3	96.9 ± 27.9
HDL-cholesterol, early pregnancy (mg/dL)	70.8 ± 15.9	65.7 ± 14.2	64.4 ± 13.8 *	67.7 ± 15.2
FPG, early pregnancy (mg/dL)	81.1 ± 5.7	82.5 ± 5.5	85.6 ± 7.0 *^,†^	87.2 ± 8.4 *^,†^
HbA1c, early pregnancy (%)	4.99 ± 0.29	5.06 ± 0.29	5.11 ± 0.29 *	5.25 ± 0.31 *^,†,§^
HbA1c, early pregnancy (mmol/mol)	31.0 ± 3.2	31.8 ± 3.1	32.3 ± 3.2 *	33.9 ± 3.4 *^,†,§^
Fasting insulin, early pregnancy (µU/mL)	7.5 (5.3–10.2)	9.2 (6.1–14.7)	10.8 (7.9–14.2) *	10.9 (8.0–17.3) *
Fasting C-Peptide, early pregnancy (ng/mL)	1.50 (1.20–1.90)	1.85 (1.50–2.30) *	1.90 (1.70–2.30) *	2.10 (1.60–7.20) *
QUICKIi, early pregnancy (dimensionless) × 10^2^	36.2 ± 3.3	35.4 ± 3.9	34.2 ± 3.2 *	33.6 ± 3.0 *^,†^
QUICKIc, early pregnancy (dimensionless) × 10^2^	48.0 ± 3.8	46.2 ± 3.8 *	45.3 ± 3.3 *	44.9 ± 3.8 *
IGI, early pregnancy (ng/mg)	2.0 ± 0.75	2.4 ± 1.0 *	2.4 ± 0.7 *	2.5 ± 0.8 *
DI, early pregnancy (ng mg^−1^ (µU/mL)^−1^) × 10^2^	25.0 (20.8–30.7)	23.3 (19.1–28.7)	21.2 (17.3–25.6) *	20.9 (17.1–24.7) *
OGTT Glucose 0 min (mg/dL)	79.2 ± 6.7	80.5 ± 6.5	91.1 ± 10.1 *^,†^	92.5 ± 14.1 *^,†^
OGTT Glucose 60 min (mg/dL)	122.9 ± 26.9	134.1 ± 26.6 *	171.7 ± 29.4 *^,†^	182.4 ± 34.5 *^,†^
OGTT Glucose 120 min (mg/dL)	101.5 ± 19.1	106.9 ± 21.9	131.0 ± 30.4 *^,†^	135.5 ± 32.4 *^,†^

Data are the mean ± SD or median (IQR) and count (%) for women with normal glucose tolerance (NGT) or gestational diabetes mellitus (GDM) in the current pregnancy stratified according to the history of GDM at previous gestation: negative history of a pregnancy with GDM (pGDM [−]), history of GDM in previous pregnancy (pGDM [+]). BMI: body mass index; FPG: fasting plasma glucose; HbA1c: glycated hemoglobin A1c; QUICKIi: quantitative insulin sensitivity check index from insulin (QUICKIi) and C-peptide (QUICKIc); IGI: insulinogenic index; DI: disposition index. * *p* < 0.05 vs. NGT-pGDM [−]. ^†^ *p* < 0.05 vs. NGT-pGDM [+]. ^§^ *p* < 0.05 vs. GDM-pGDM [−].

**Table 3:** Assessment of possible predictors for GDM in current pregnancy for patients with history of GDM.

GDM	OR	95% CI	*p*-Value	C-Index
Age (years)	1.04	0.96–1.12	0.360	55.0
Parity (≥2)	1.09	0.52–2.29	0.811	57.9
Previous birth weight (>4000 g)	1.52	0.57–4.34	0.412	52.8
Previous birth weight (>90 Pct)	1.34	0.58–3.23	0.501	52.7
Time between pregnancies (years)	0.98	0.89–1.07	0.639	56.9
No. pregnancies with GDM (>1)	1.56	0.62–4.18	0.360	56.9
History of insulin treatment	2.07	1.00–4.39	0.053	58.9
Ethnicity (non Caucasian)	1.82	0.82–4.21	0.150	56.1
Family history with diabetes (1st or 2nd degree)	0.97	0.46–2.02	0.930	49.6
BMI, current (kg/m^2^)	1.05	0.99–1.13	0.129	57.9
BMI, before pregnancy (kg/m^2^)	1.04	0.98–1.11	0.202	56.1
FPG, early pregnancy (mg/dL)	1.10	1.04–1.18	0.002	66.8
HbA1c, early pregnancy (mmol/L)	1.21	1.08–1.39	0.002	66.7
Fasting insulin, early pregnancy (µU/mL)	1.07	1.01–1.14	0.027	61.6
Fasting C-Peptide, early pregnancy (ng/mL)	1.38	0.86–2.33	0.200	58.7
QUICKIi, early pregnancy (dimensionless) × 10^2^	0.86	0.76–0.96	0.011	62.4
QUICKIc, early pregnancy (dimensionless) × 10^2^	0.91	0.82–1.01	0.079	61.4
IGI, early pregnancy (ng/mg)	1.10	0.72–1.70	0.671	53.7
DI, early pregnancy (ng mg^−1^ (µU/mL)^−1^) × 10^2^	0.94	0.88–0.98	0.017	63.0
Total-cholesterol, early pregnancy (mg/dL)	1.00	0.99–1.01	0.767	47.2
LDL-cholesterol, early pregnancy (mg/dL)	0.99	0.98–1.01	0.397	55.6
HDL-cholesterol, early pregnancy (mg/dL)	1.00	0.98–1.04	0.477	51.4
Triglycerides, early pregnancy (mg/dL)	1.00	0.99–1.01	0.585	52.3

Data are odds ratio (OR) and 95% confidence interval for the association with the development of GDM in the current pregnancy for women with history of GDM in previous gestation. BMI, body mass index; FPG, fasting plasma glucose; HbA1c, glycated haemoglobin A1c; QUICKIi, quantitative insulin sensitivity check index from insulin (QUICKIi) and C-peptide (QUICKIc); IGI, insulinogenic index; DI, disposition index.

## Data Availability

The data that support the findings of this study are available from the authors, G.K. and C.S.G. upon reasonable request.

## Supplementary Materials

The following are available online at https://www.mdpi.com/article/10.3390/jcm10204794/s1, Table S1: Characteristics of the study sample after excluding study participants, who had their index pregnancy before the implementation of universal OGTT testing in Austria, Table S2: Pregnancy outcome and offspring data (multiple pregnancies and cases with missing pregnancy outcome data are excluded).
