# Supplementary material for: Recurrence of Gestational Diabetes Mellitus: To Assess Glucose Metabolism and Clinical Risk Factors at the Beginning of a Subsequent Pregnancy
Source: J Clin Med. 2021 Oct 19;10(20):4794. doi: 10.3390/jcm10204794 (PMC8540668; doi:10.3390/jcm10204794)
Supplement: Supplementary file 1 [file jcm-10-04794-s001.zip › jcm-1392018-supplementary.pdf]

Supplemental Material

Table S1. Characteristics of the study sample after excluding study participants, who had their index pregnancy before the implementation of universal OGTT testing in Austria.

Variable	pGDM [–] (*n* = 451)	pGDM [+] (*n* = 105)	*p*-Value
Age (years)	31.5 ± 5.2	32.4 ± 4.6	0.083
Parity (≥2)	190 (42.1)	62 (59.0)	0.002
Parity (≥3)	75 (16.6)	25 (23.8)	0.086
Time between pregnancies (years)	3.0 (2.0–4.0)	3.0 (2.0–5.0)	0.108
Ethnicity (non Caucasian)	117 (25.9)	33 (31.4)	0.255
Family history (1st and 2nd degree)	196 (43.5)	62 (59.0)	0.004
BMI, current (kg/m^2)	25.6 ± 5.5	28.3 ± 5.7	<0.001
BMI, before pregnancy (kg/m^2)	25.1 ± 5.4	27.7 ± 5.8	<0.001
Multiple pregnancy	40 (8.9)	3 (2.9)	0.049
Triglycerides, early pregnancy (mg/dL)	114 ± 43.5	130 ± 46.8	0.003
Total-cholesterol, early pregnancy (mg/dL)	187 ± 34.8	193 ± 32.2	0.091
LDL-cholesterol, early pregnancy (mg/dL)	95.0 ± 28.2	101.1 ± 26.5	0.040
HDL-cholesterol, early pregnancy (mg/dL)	69.1 ± 14.7	66.3 ± 14.4	0.089
FPG, early pregnancy (mg/dL)	81.6 ± 6.0	85.1 ± 7.6	<0.001
HbA1c, early pregnancy (%)	5.00 ± 0.29	5.16 ± 0.32	<0.001
HbA1c, early pregnancy (mmol/moL)	31.1 ± 3.2	32.9 ± 3.5	<0.001
Fasting insulin, early pregnancy (µU/mL)	7.7 (5.4–10.6)	10.7 (6.9–16.6)	<0.001
Fasting C-Peptide, early pregnancy (ng/mL)	1.50 (1.28–1.90)	2.00 (1.50–2.58)	<0.001
QUICKIi, early pregnancy (dimensionless) × 10^2	36.0 ± 3.4	34.3 ± 3.6	<0.001
QUICKIc, early pregnancy (dimensionless) × 10^2	47.8 ± 3.8	45.4 ± 3.9	<0.001
IGI, early pregnancy (ng/mg)	2.0 ± 0.72	2.4 ± 0.91	<0.001
DI, early pregnancy (ng mg^{-1} (µU/mL)$^{-1}$) × 10^2	24.7 (20.3–30.4)	21.3 (17.5–26.4)	<0.001
GDM, current pregnancy	66 (14.6)	59 (56.2)	<0.001
OGTT Glucose 0 min (mg/dL)	80.7 ± 8.3	86.4 ± 9.7	<0.001
OGTT Glucose 60 min (mg/dL)	128.5 ± 32.3	158.0 ± 40.6	<0.001
OGTT Glucose 120 min (mg/dL)	104.3 ± 22.5	121.6 ± 32.7	<0.001

Data are mean ± SD or median (IQR) and count (%) for women without history of a pregnancy with GDM (pGDM [−]) vs. patients with history of GDM in previous pregnancy (pGDM [+]). BMI, body mass index; FPG, fasting plasma glucose; HbA1c, glycated haemoglobin A1c; QUICKIi, quantitative insulin sensitivity check index from insulin (QUICKIi) and C-peptide (QUICKIc); IGI, insulinogenic index; DI, disposition index.

Table S2. Pregnancy outcome and offspring data (multiple pregnancies and cases with missing pregnancy outcome data are excluded).

Variable	pGDM [−] (n = 536)	pGDM [+] (n = 117)	p-Value
Induction of fetal lung maturation	31 (5.9)	3 (2.6)	0.164
Cesarean section	233 (44.5)	52 (45.2)	0.883
Vacuum extraction	13 (2.5)	3 (2.6)	0.946
Neonatal intensive care unit admission	30 (5.8)	3 (2.7)	0.193
GAD (weeks)	39.0 (38.0–40.0)	38.5 (38.0–40.0)	0.844
Preterm delivery (<37 weeks)	42 (8.0)	7 (6.0)	0.469
Birth weight (percentile)	45.8 ± 27.0	51.8 ± 27.4	0.037
Birth length (percentile)	44.8 ± 27.8	47.0 ± 29.1	0.460
LGA	28 (5.4)	11 (9.9)	0.078

Data are mean ± SD or median (IQR) and count (%) for women without history of a pregnancy with GDM (pGDM [−]) vs. patients with history of GDM in previous pregnancy (pGDM [+]). GAD, gestational age at delivery; LGA, large for gestational age offspring.
